# Occupation and Educational Attainment Characteristics Associated With COVID-19 Mortality by Race and Ethnicity in California

**DOI:** 10.1001/jamanetworkopen.2022.8406

**Published:** 2022-04-22

**Authors:** Ellicott C. Matthay, Kate A. Duchowny, Alicia R. Riley, Marilyn D. Thomas, Yea-Hung Chen, Kirsten Bibbins-Domingo, M. Maria Glymour

**Affiliations:** 1 Center for Health and Community, University of California, San Francisco; 2 Department of Epidemiology and Biostatistics, University of California, San Francisco; 3 Department of Sociology, University of California, Santa Cruz

## Abstract

**Importance:**

Racial and ethnic inequities in COVID-19 mortality may be driven by occupation and education, but limited evidence has assessed these mechanisms.

**Objective:**

To estimate whether occupational characteristics or educational attainment explained the associations between race and ethnicity and COVID-19 mortality.

**Design, Setting, and Participants:**

This population-based retrospective cohort study of Californians aged 18 to 65 years linked COVID-19 deaths to population estimates within strata defined by race and ethnicity, gender, age, nativity in the US, region of residence, education, and occupation. Analysis was conducted from September 2020 to February 2022.

**Exposures:**

Education and occupational characteristics associated with COVID-19 exposure (essential sector, telework option, wages).

**Main Outcomes and Measures:**

All confirmed COVID-19 deaths in California through February 12, 2021. The study estimated what COVID-19 mortality would have been if each racial and ethnic group had (1) the COVID-19 mortality risk associated with the education and occupation distribution of White people and (2) the COVID-19 mortality risk associated with the lowest-risk educational and occupational positions.

**Results:**

Of 25 235 092 participants (mean [SD] age, 40 [14] years; 12 730 395 [50%] men), 14 783 died of COVID-19, 8 125 565 (32%) had a Bachelor’s degree or higher, 13 345 829 (53%) worked in essential sectors, 11 783 017 (47%) could not telework, and 12 812 095 (51%) had annual wages under $51 700. COVID-19 mortality ranged from 15 deaths per 100 000 for White women and Asian women to 139 deaths per 100 000 for Latinx men. Accounting for differences in age, nativity, and region of residence, if all races and ethnicities had the COVID-19 mortality associated with the occupational characteristics of White people (sector, telework, wages), COVID-19 mortality would be reduced by 10% (95% CI, 6% to 14%) for Latinx men, but increased by 5% (95% CI, −8% to 17%) for Black men. If all working-age Californians had the COVID-19 mortality associated with the lowest-risk educational and occupational position (Bachelor’s degree, nonessential, telework, and highest wage quintile), there would have been 43% fewer COVID-19 deaths among working-age adults (8441 fewer deaths; 95% CI, 32%-54%), with the largest absolute risk reductions for Latinx men (3755 deaths averted; 95% CI, 3304-4255 deaths) and Latinx women (2329 deaths averted; 95% CI, 2038-2621 deaths).

**Conclusions and Relevance:**

In this population-based cohort study of working-age California adults, occupational disadvantage was associated with excess COVID-19 mortality for Latinx men. For all racial and ethnic groups, excess risk associated with low-education, essential, on-site, and low-wage jobs accounted for a substantial fraction of COVID-19 mortality.

## Key Points

**Question:**

To what extent are inequities in educational attainment and occupational characteristics associated with racial and ethnic inequities in COVID-19 mortality?

**Findings:**

In this cohort study of 25 million working-age adults in California, differences in the distribution of education and occupation across racial and ethnic groups were associated with racial and ethnic inequities in COVID-19 mortality, particularly for Latinx adults. If every working-age Californian had the COVID-19 mortality risk associated with the lowest-risk educational and occupational position, there would have been an estimated 8441 (43%) fewer deaths in this population.

**Meaning:**

Educational and occupational disadvantage are important factors associated with risk for COVID-19 mortality, but eliminating avoidable excess risk associated with low-education, essential, on-site, and low-wage jobs is unlikely to be sufficient alone to achieve equity.

## Teaser

This cohort study evaluates the association between occupation and COVID-19 mortality by race and ethnicity in California.

## Introduction

As of July 16, 2021, individuals identifying as Latinx were 2.3 times more likely to have died from COVID-19 than non-Latinx White persons in the US.^1^ Inequities for Black and American Indian or Alaska Native groups were similarly large.^1^ Research to date probing the drivers of racial and ethnic inequities in COVID-19 outcomes has focused primarily on factors such as age, gender, health care access, and comorbidities, all of which inadequately explain racial and ethnic differences in COVID-19 cases, severe morbidity, and mortality.^2,3,4,5^

One potential factor associated with racial and ethnic inequities in COVID-19 outcomes is the unequal distribution of occupations across racial and ethnic groups.^6,7^ During the COVID-19 pandemic, Black and Latinx workers were substantially more likely than White and Asian workers to hold low-wage, essential jobs and jobs with the greatest risk of SARS-CoV-2 exposure relative to population size.^8^ In turn, several studies have documented wide differences in overall rates of COVID-19 infection or death by occupational factors.^9,10,11,12,13^ Ecological studies^14,15,16^ suggest that racial and ethnic inequities in COVID-19 outcomes are associated with composition of worker types in communities. One study found that Black and Latinx adults with health conditions that place them at high risk for severe COVID-19 illness were more likely to live in households with essential workers and workers whose jobs cannot be done from home, but actual COVID-19 outcomes were not evaluated.^17^ Workplace outbreaks of COVID-19 also appear to disproportionately affect workers from racial and ethnic minority groups.^18^ Yet individual-level research is needed that quantifies the association between occupational characteristics specific to COVID-19 and racial and ethnic inequities in COVID-19 outcomes. Moreover, structural racism affects the educational opportunities that determine job opportunities^19^ and shapes capacities of employees to negotiate for sick leave, access to protective equipment, and other workplace safety conditions.^20^ COVID-19 mortality differs by education within occupational strata,^21^ but the joint role of education and occupation in racial and ethnic inequities in COVID-19 mortality is unknown.

We examined the extent to which racial and ethnic inequities in COVID-19 mortality among the California working-age population were associated with differences in education and occupations.^22^ We estimated the magnitude of inequities that would remain if all racial and ethnic groups had the COVID-19 mortality risk of those with low-risk educational and occupational positions.

## Methods

We followed the Strengthening the Reporting of Observational Studies in Epidemiology (STROBE) reporting guideline for cohort studies. This study was approved by the institutional review boards of the California Department of Public Health and the University of California, San Francisco. No informed consent was required because the study involved only decedents and publicly available data, in accordance with 45 CFR §46.

### Death Data and Measures

From California death records, we identified all confirmed COVID-19 deaths occurring between January 1, 2020, and February 12, 2021. Records included the decedent’s race and ethnicity, gender, date of birth and death, educational attainment, place of birth, place of residence, and open text fields for primary occupation and industry, described as “work done during most of working life.” COVID-19 deaths were those with a primary *International Classification of Diseases, Tenth Revision *diagnostic code of U071.

We conceptualized race and ethnicity as socially defined categories that govern the distribution of risk, opportunities, and discrimination.^23^ We classified race and ethnicity as Asian, Black, Latinx, White, and other (including American Indian, Alaskan Native, Native Hawaiian, other Pacific Islander, multiracial, and unspecified). Apart from Latinx, all racial and ethnic groups were non-Latinx. We used the National Institute for Occupational Safety and Health’s Industry and Occupation Computerized Coding System, an automated machine learning-based system, to convert the occupation and industry text to standardized 2010 Census codes. Educational attainment categories were no high school degree and no GED; high school degree or GED; some college or Associate’s degree; and Bachelor’s degree or beyond. We considered US born vs born outside of the US status because, compared with US born individuals, individuals born outside of the US are more likely to hold essential jobs and have experienced higher COVID-19 mortality in California.^21^ Individuals born outside of the US who are undocumented may encounter labor market discrimination or have less power to select or negotiate for safe working conditions. Place of residence may affect both the occupations available and COVID-19 mortality,^22^ and was grouped into 10 California regions (eTable 1 in the Supplement). Given our focus on workers, we restricted decedents to those aged 18 to 65 years, in alignment with prior research.^9^ Age groups were 18 to 24 years, 5-year age groups between ages 25 and 59 years, and 60 to 65 years.

We defined population strata by cross-classifying all categories of all variables selected from the death records. The cross-classification of race and ethnicity, gender, age group, nativity, region, education, and occupational category created 3 672 000 total possible strata, of which 12 850 were represented in the death data. We then created a data set composed of stratum-level COVID-19 death counts by summing the number of COVID-19 deaths in each stratum.

### Population Data and Measures

To characterize the population at risk of death, we used the 2019 American Community Survey (ACS) California person-level microdata. We defined strata using the same set of variables as in the death records. Restricting to the same ages (18-65 years), we created population counts by summing the ACS person weights representing the number of people in each stratum (174 315 strata had nonzero populations).

### Occupational Characteristics

We characterized occupations using multiple measures hypothesized to be associated with SARS-CoV-2 exposure risk (eAppendix 1 and eTable 2 in the Supplement). First, we categorized the 529 unique 2010 US Census occupation codes into 9 occupational sectors based on the California official definition of essential work^24^ using a crosswalk established in previous research^9^: facilities; food or agriculture; government or community; health or emergency; manufacturing; retail; transportation or logistics; not essential; and unemployed, not in labor force, or missing.

Second, we linked occupation codes to an established classification of which jobs can be done at home during the pandemic, based on a composite of job characteristics measured in the O*NET database. In secondary analyses, we considered 13 individual O*NET measures (eAppendix 1 in the Supplement).

Third, we linked occupation codes to their median annual wages reported by the Bureau of Labor Statistics. Individuals with lower incomes may have less ability to forgo work or income when faced with undesired COVID-19 exposure risk. In secondary analyses, we considered other quantiles of wages. To merge the telework, O*NET, and wages measures with the death and population data, we used multiple occupation code crosswalks (eAppendix 2 in the Supplement).

### Construction of Analytical Data Set

We appended (ie, stacked) the death and ACS population data to create 1 strata-level data set representative of the cohort of all working-age Californians. The outcome was COVID-19 death: rows derived from the death data were assigned a 1, and rows derived from the ACS data were assigned a 0. Each row had a weight corresponding to the number of individuals represented. These weights were included in all statistical analyses. Because the ACS does not indicate which individuals died of COVID-19, this data structure implies that the fewer than 0.1% of Californians aged 18 to 65 years who died of COVID-19 are represented twice. Records with missing educational or occupational measures were excluded (<1% of participants). The analytical data set included 187 165 rows representing 25 235 092 individuals.

### Statistical Analysis

We fit a sequence of linear probability models^25^ and used these to estimate the COVID-19 mortality risk for each racial and ethnic group under 3 hypothetical distributions of covariates and educational and occupational characteristics.^26^ First, under the composition-adjusted scenario, we estimated the COVID-19 mortality risk if all racial and ethnic groups had the same distribution of covariates (age, nativity, and region) as White individuals, using linear regression of COVID-19 death on race and ethnicity adjusted for covariates. Second, under the composition-adjusted and education- or occupation-adjusted scenario, we estimated the COVID-19 mortality risk if all racial and ethnic groups had the same distribution of covariates and educational and occupational position(s) as White individuals, using linear regression of COVID-19 death on race and ethnicity adjusted for covariates and the hypothesized factor(s) associated with racial and ethnic inequities in COVID-19 outcomes (education, essential sector, telework, wages, the 3 occupational measures simultaneously, or all 4 measures simultaneously). Third, under the composition-adjusted with lowest-risk education and occupation scenario, we estimated the COVID-19 mortality risk if all racial and ethnic groups had the same distribution of covariates as White individuals and if all individuals, regardless of race and ethnicity, were in the lowest-risk categories of education and occupation (ie, Bachelor’s degree or higher, nonessential, telework, highest quintile of wages), using the same models as in the second scenario.

Because both the occupations available and occupational risk differ for men and women within racial and ethnic groups, we stratified all analyses by gender.^27,28^ To allow for interactions and nonlinear associations, we converted all continuous measures to quintile-based categorical measures and included all possible first-order interaction terms. For occupational factors, nonworkers (16% of participants) were included as a distinct category.

To estimate potential deaths averted, we multiplied estimated risk differences by corresponding population counts. We generated 95% CIs using the nonparametric bootstrap (eAppendix 3 in the Supplement).^29^ Because linear probability models can estimate risks outside of the range 0 to 1, we conducted sensitivity analyses using logistic regression. Statistical analyses were completed in R statistical software version 4.0.2 (R Project for Statistical Computing) from September 2020 to February 2022.

## Results

The total California population in the study was 25 249 875. Individuals had a mean (SD) age of 40 (14) years, and 12 730 395 (50%) were men. Among Californians aged 18 to 65 years, 14 783 deaths were attributed to COVID-19 between January 1, 2020, and February 12, 2021 (risk, 59 deaths per 100 000 persons). By design, the demographics of the study population matched the 2019 California population (Table 1). A total of 8 125 565 individuals (32%) had a Bachelor’s degree or higher, 53% (13 345 829 individuals) worked in essential sectors, 11 783 017 individuals (47%) could not telework, and 12 812 095 (51%) had annual wages under $51 700. COVID-19 mortality ranged from 15 deaths per 100 000 for White women and Asian women to 139 deaths per 100 000 for Latinx men. Compared with the general population, COVID-19 decedents were disproportionately older, men, and Latinx, with lower education and occupational positions (Table 1). Individuals with a high school education or less composed 36% (9 238 193 individuals) of the study population but 69% (10 191 individuals) of COVID-19 deaths; individuals holding jobs amenable to telework composed 36% (8 960 489 individuals) of the population but only 18% (2681 individuals) of COVID-19 deaths.

**Table 1.** Demographic, Educational, and Occupational Characteristics of 2019 California Population and California COVID-19 Decedents Ages 18 to 65 Years, January 1, 2020, to February 12, 2021

Characteristic	Individuals, No. (%)	
	California population (N = 25 235 092)	California COVID-19 deaths (n = 14 783)
Age, y		
18-24	3 688 420 (15)	82 (1)
25-44	11 347 306 (45)	1852 (13)
45-65	10 199 366 (40)	12 849 (87)
Gender		
Women	12 514 931 (50)	4549 (31)
Men	12 720 161 (50)	10 234 (69)
Race and ethnicity		
American Indian or Alaskan Native	93 822 (<1)	96 (1)
Asian	3 925 494 (16)	1118 (8)
Black	1 472 151 (6)	966 (7)
Latinx	9 859 259 (39)	10 229 (69)
Native Hawaiian and other Pacific Islander	100 949 (<1)	134 (1)
Other	937 809 (4)	472 (3)
Some other race or ethnicity^a^	68 339 (<1)	108 (1)
≥2 Races	674 227 (3)	107 (1)
Unknown	472 (<1)	27 (<1)
White	9 040 379 (36)	1998 (14)
Born outside of the US		
Yes	8 305 063 (33)	8808 (60)
No	16 929 792 (67)	5738 (39)
Missing	237 (0)	237 (2)
Educational attainment		
No high school degree and no GED	3 633 198 (14)	5377 (36)
High school degree or GED	5 594 804 (22)	4814 (33)
Some college or Associate’s degree	7 882 332 (31)	2595 (18)
Bachelor’s degree or higher	8 124 163 (32)	1402 (9)
Missing	595 (<1)	595 (4)
Worker sector		
Facilities	2 561 383 (10)	2715 (18)
Food and agriculture	1 888 544 (7)	1504 (10)
Government and community	2 313 800 (9)	920 (6)
Health or emergency	2 025 529 (8)	1006 (7)
Manufacturing	1 157 573 (5)	1394 (9)
Retail	1 583 633 (6)	698 (5)
Transportation and logistics	1 805 127 (7)	2003 (14)
Not essential	7 805 001 (31)	2029 (14)
Unemployed or not in labor force	4 094 502 (16)	1745 (12)
Missing	0	769 (5)
Telework-amenable occupation		
Yes	8 957 808 (35)	2681 (18)
No	11 773 666 (47)	9351 (63)
Unemployed or not in labor force	4 094 495 (16)	2507 (17)
Missing	409 123 (2)	244 (2)
Median annual wage for occupation		
$22 200-29 000	4 722 209 (19)	3003 (20)
$29 001-39 100	4 130 764 (16)	3405 (23)
$39 101-51 700	3 949 646 (16)	3068 (21)
$51 701-73 800	3 969 152 (16)	1677 (11)
≥$73 800	4 196 965 (17)	1068 (7)
Unemployed or not in labor force	4 094 495 (16)	2507 (17)
Missing	171 861 (1)	55 (<1)

^a^ “Some other race or ethnicity” includes all other races and ethnicities for which there were no specific response options provided in the American Community Survey or death records.

In unadjusted analyses, Latinx, Black, and other minoritized race and ethnicity groups had greater COVID-19 mortality than White people of the same gender (Table 2). Gaps vs White people were particularly pronounced for Latinx people. For Asian women, COVID-19 mortality matched that of White women. Large differences in mortality persisted after accounting for compositional differences in age, nativity, and region of residence.

**Table 2.** Estimated COVID-19 Mortality Risks After Accounting for Racial and Ethnic Differences in Composition, Education, and Occupational Characteristics, for Individuals Aged 18 to 65 Years, by Race, Ethnicity, and Gender, California, January 1, 2020, to February 12, 2021

Gender and race or ethnicity	Unadjusted deaths per 100 000 persons	Adjusted deaths per 100 000 persons (95% CI)						
		Composition^a^	Composition and work sector^a^	Composition and telework^a^	Composition and wages^a^	Composition and education^a^	Composition and all occupational characteristics ^a^	Composition, education, and all occupational characteristics ^a^
Women								
Asian	15	14 (10-18)	13 (9-17)	13 (9-16)	14 (11-17)	17 (13-21)	12 (9-16)	16 (12-20)
Black	48	51 (44-58)	52 (45-59)	52 (45-59)	53 (46-59)	47 (40-54)	51 (43-59)	47 (40-55)
Latinx	60	70 (66-74)	69 (65-72)	68 (64-72)	66 (63-70)	55 (51-58)	67 (63-71)	54 (51-57)
White	15	15 [Reference]	15 [Reference]	15 [Reference]	15 [Reference]	15 [Reference]	15 [Reference]	15 [Reference]
Other^b^	29	46 (36-52)	44 (36-52)	45 (36-54)	43 (35-52)	41 (33-48)	45 (37-53)	42 (33-50)
Men								
Asian	41	28 (21-34)	27 (20-34)	26 (20-32)	27 (21-33)	34 (27-40)	27 (21-33)	33 (26-40)
Black	77	87 (79-94)	96 (87-104)	96 (88-104)	88 (80-97)	77 (69-85)	91 (80-102)	86 (78-95)
Latinx	138	140 (135-145)	127 (122-132)	132 (127-138)	128 (123-134)	116 (111-121)	127 (121-132)	110 (105-116)
White	26	26 [Reference]	26 [Reference]	26 [Reference]	26 [Reference]	26 [Reference]	26 [Reference]	26 [Reference]
Other^b^	57	83 (72-94)	81 (70-92)	81 (71-91)	80 (70-91)	77 (67-87)	81 (69-94)	80 (69-91)

^a^ Composition-adjusted COVID-19 mortality risks indicate the estimated COVID-19 mortality risk if all racial and ethnic groups had the same distribution of age, nativity, and region of residence as White people. Composition- and education-, work sector-, telework-, or wages-adjusted COVID-19 mortality risks indicate the estimated COVID-19 mortality risk if all racial and ethnic groups had the same distribution of age, nativity, region of residence, and educational attainment, work sector, telework capacity, or wages as White people, respectively.

^b^ Other race and ethnicity includes American Indian, Alaskan Native, Native Hawaiians, other Pacific Islanders, multiracial, and unspecified (all non-Latinx).

Table 2 presents adjusted COVID-19 mortality if all groups had the COVID-19 mortality risk associated with the occupation or education distribution of White people of the same gender (for risk differences, see eTable 3 in the Supplement). When equalizing the distribution of work sector or telework capacity across racial and ethnic groups, we estimated little change in COVID-19 mortality for women, but reduced mortality for Latinx men (6%-9%) and increased mortality for Black men (10%). The latter finding is consistent with the pattern that Black people aged 18 to 65 years in California on average hold fewer nonessential jobs than White people (386 578 [26%] vs 3 582 906 [40%]) and are more likely to be unemployed or not in the labor force (332 118 [23%] vs 1 263 136 [14%]) (eTable 4 in the Supplement), which is associated with lower COVID-19 mortality (Table 1). When equalizing COVID-19 mortality risk associated with differing wage levels, COVID-19 mortality was reduced for Latinx women and men and other race and ethnicity women, but there was little change for other groups. Equalizing all occupational characteristics simultaneously (work sector, telework, and wages) reduced COVID-19 mortality by 10% for Latinx men (95% CI: 6% to 14%), increased COVID-19 mortality by 5% for Black men (95% CI: −8% to 17%) and had little impact on COVID-19 mortality for other groups (eTable 5 in the Supplement).

If all groups had the COVID-19 mortality risk associated with the educational attainment of White people of the same gender, we estimated that COVID-19 mortality would be reduced (and therefore more equal) for Latinx women (22%) and men (17%), Black women (8%) and men (11%), and other race and ethnicity women (8%) and men (7%) (Table 2). However, COVID-19 mortality inequalities would be exacerbated for Asian women (21%) and men (21%), reflecting that on average Asian people in California have more education than White people (eTable 4 in the Supplement). If each group had the COVID-19 mortality associated with the education, essential sector, telework, and wage distribution of White people, COVID-19 mortality would be reduced by 23% for Latinx women, 21% for Latinx men, and smaller amounts for Black women and other race and ethnicity individuals, but increased by 14% for Asian women and 20% for Asian men. Sensitivity analyses using logistic regression were consistent with the primary findings (eTable 6 in the Supplement).

If all groups had the COVID-19 mortality associated with the lowest-risk educational and occupational positions (Bachelor’s degree or higher, nonessential, occupation with telework available, highest quintile of median annual wages), we estimated that COVID-19 mortality would be substantially reduced for all groups (Figure), with reductions ranging from 4 deaths per 100 000 among Black women to 75 deaths per 100 000 among Latinx men.

**Figure.**
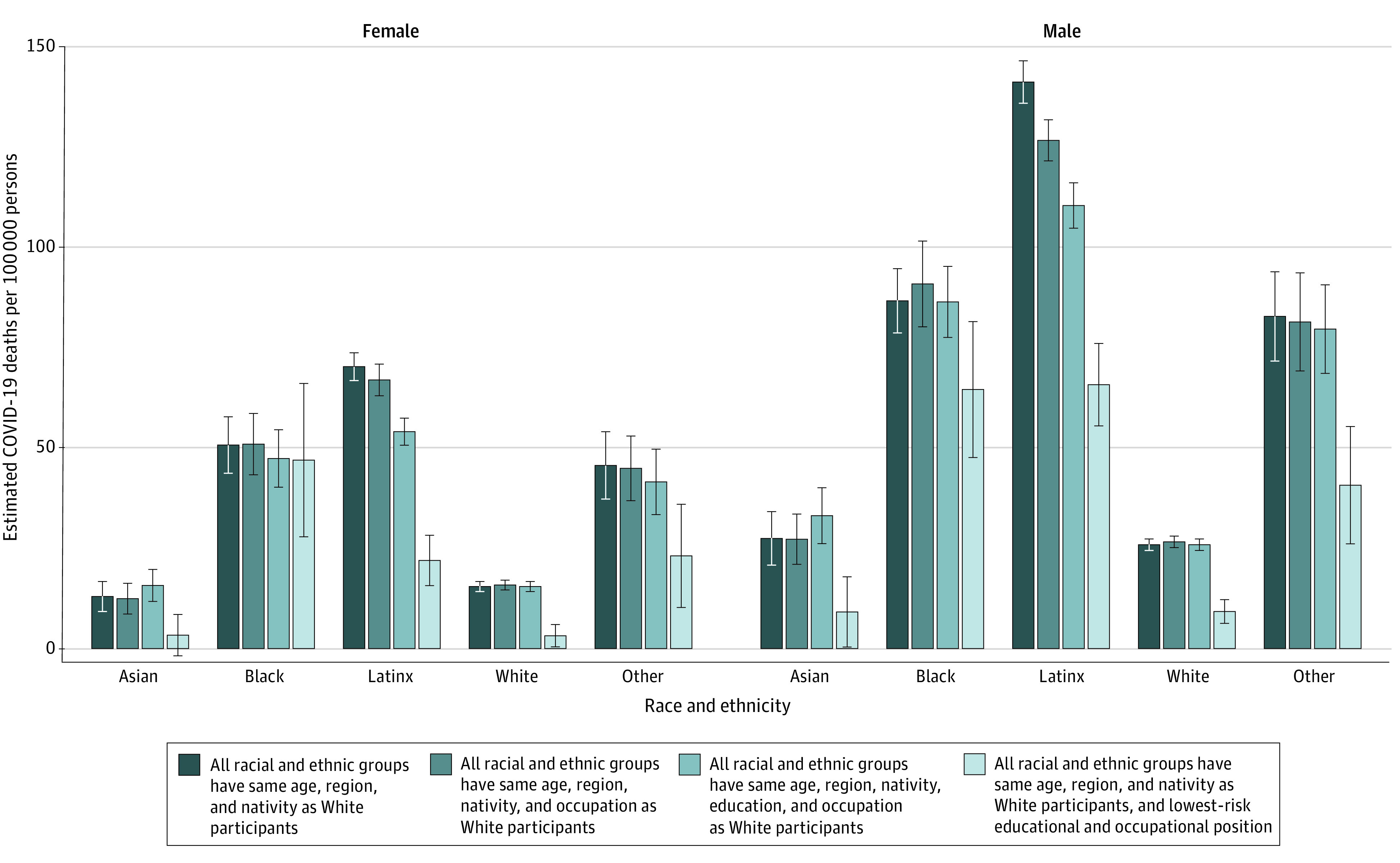
Estimated COVID-19 Mortality for Individuals Aged 18 to 65 Years, by Race, Ethnicity, and Gender, Under Alternative Compositional, Educational, and Occupational Distributions, California, January 1, 2020, to February 12, 2021 Error bars indicate 95% CIs. Other race and ethnicity includes American Indian, Alaskan Native, Native Hawaiian, other Pacific Islander, multiracial, and unspecified (all non-Latinx). Estimates present the estimated COVID-19 mortality risk for each racial or ethnic–gender group under 4 scenarios. Lowest-risk educational and occupational position indicates holding a Bachelor's degree or higher and working in a nonessential telework-amenable occupation in the highest quintile of median annual wages.

If every working-age Californian had had the COVID-19 mortality risk associated with the lowest-risk education and occupational position, we estimate that there would have been substantially fewer COVID-19 deaths for all groups (Table 3). Overall, this shift was associated with 8441 COVID-19 deaths averted (95% CI, 6790-10 096 deaths), a 43% reduction (95%, CI, 32%-54%). The vast majority of this absolute risk reduction was among Latinx men (3755 potential deaths averted [95% CI, 3304-4255 deaths]) and women (2329 potential deaths averted [95% CI, 2038-2621 deaths]).

**Table 3.** Estimated COVID-19 Mortality Reductions and Potential Deaths Averted, for Individuals Aged 18-65 Years, per 100 000 Persons, by Race or Ethnicity and Gender, If All Groups Held the Lowest-Risk Educational and Occupational Positions, California, January 1, 2020, to February 12, 2021

Gender and race or ethnicity	Population	COVID-19 mortality risk per 100 000 if all individuals had the lowest risk (95% CI)	Change relative to composition-adjusted, %	Absolute risk reduction per 100 000, in lowest risk scenario (95% CI)	Estimated potential deaths averted in lowest risk scenario (95% CI)^a^
Women					
Asian	2 063 142	3 (0 to 6)	−77	−10 (−14 to −5)	206 (103 to 289)
Black	723 166	47 (28 to 66)	−8	−4 (−22 to 14)	29 (−101 to 159)
Latinx	4 852 961	22 (16 to 28)	−69	−48 (−54 to −42)	2329 (2038 to 2621)
White	4 407 412	3 (0 to 6)	−80	−12 (−15 to −9)	529 (397 to 661)
Other^b^	468 250	23 (10 to 36)	−50	−23 (−35 to −10)	108 (47 164)
Men					
Asian	1 862 352	9 (0 to 18)	−67	−18 (−25 to −12)	335 (223 to 466)
Black	748 985	65 (48 to 81)	−25	−22 (−39 to −6)	165 (45 to 292)
Latinx	5 006 298	66 (55 to 76)	−53	−75 (−85 to −66)	3755 (3304 to 4255)
White	4 632 967	9 (6 to 12)	−65	−17 (−20 to −13)	788 (602 to 927)
Other^b^	469 559	41 (26 to 55)	−51	−42 (−56 to −28)	197 (131 to 263)

^a^ Estimates present the difference in the projected COVID-19 mortality risk for each racial and ethnic–gender group if all racial and ethnic groups had the same distribution of age, nativity, and region of residence as White people vs if all racial and ethnic groups additionally were in the lowest-risk education and occupation positions (had a Bachelor’s degree or higher and worked in nonessential, telework-amenable occupations in the highest quintile of median annual wages).

^b^ Other race and ethnicity includes American Indian, Alaskan Native, Native Hawaiians, other Pacific Islanders, multiracial, and unspecified (all non-Latinx).

## Discussion

In this population-based analysis of working-age Californians, large racial and ethnic inequities in COVID-19 mortality were associated with differences in educational attainment and measured occupational characteristics. The fraction of inequality in comparison to White people that was associated with occupation varied by race and ethnicity and gender and was greatest for Latinx men. Education was more associated with racial and ethnic inequities in COVID-19 mortality than measured occupational characteristics. In all racial and ethnic groups, we estimate that COVID-19 mortality would have been substantially reduced if excess risk associated with educational and occupational disadvantage were eliminated. If COVID-19 mortality were no higher in low-education, essential, on-site, or low-wage jobs than in high-education, nonessential, high-paying jobs with teleworking options, we estimated that 43% of California’s working-age COVID-19 deaths would have been prevented. In terms of absolute deaths averted, this risk reduction would confer the most benefit for Latinx men and women. Despite these reductions, substantial racial and ethnic inequities in COVID-19 mortality remained, suggesting that eliminating excess risk associated with educational and occupational disadvantage alone is insufficient to eliminate inequities.

Findings suggest that education and occupation interact to shape COVID-19 risk and inequities. Education conveys social status, social influence, and labor market resources. Education is also the leading determinant of the type and circumstances of work, so fully disentangling education from occupation is not possible. Thus, individuals with more education may be able to negotiate safer work environments or refuse to participate in unsafe workplaces.^20^ Interventions that target the education-occupation interaction might include targeting vaccine outreach to workplaces where the average worker has less than a high school degree (eg, meat-packing plants).

Consistent with other research,^30^ the estimated percentage of inequity associated with measured occupational factors was smaller than anticipated, whereas education was associated with a larger percentage of racial and ethnic inequities in COVID-19 mortality. Beyond determining work conditions and differentiating the occupational hierarchy of COVID-19 risk in ways that play a role in racial and ethnic inequities, education may also influence COVID-19 risk and inequities independent of occupation.^31,32,33^ Compared with people with more education, individuals with lower education have less access to accurate information on COVID-19 prevention, experience greater barriers to implementing recommended prevention strategies, and have less access to prevention resources through education-based social networks.^34^ Education is also associated with access to health care,^35^ which has been linked to racial and ethnic disparities in COVID-19 outcomes.^2^ These factors are all amenable to intervention.

Our findings underscore the importance of safeguarding essential workers in the highest-risk occupations by focusing on workplace interventions, such as high-quality air filtration, on-site vaccination, and free, regular rapid testing.^36^ The unequal distribution of risky work was associated with racial and ethnic mortality differences, particularly for Latinx individuals. We know how to protect workers: in California, those with high exposure risk but adequate protective policies, procedures, and equipment (eg, physicians) did not experience increases in pandemic-era mortality.^9^ This is especially important as novel pathogenic variants emerge and many remain unvaccinated: as of October 18, 2021, Latinx Californians constituted 61% of COVID-19 cases but only 35% of vaccinations received.^37^

The particular relevance of occupational factors (essential work, telework, and wages) for Latinx people may reflect occupational segregation in California. Prior research has shown that Latinx people, in particular, are overrepresented in low-wage, precarious work in the essential sectors that saw the highest COVID-19 mortality in the state.^21^ In contrast, and consistent with prior ecological research,^15,16^ our occupational measures were negligibly associated with Black-White inequities in California. Long-standing structural and interpersonal racism shapes other social factors such as residential segregation, lack of health care access, intergenerational wealth inequalities, and less-measurable variables, all of which may be associated with greater underlying risk for Black workers.

### Strengths and Limitations

Major strengths of this study are the use of population-wide death records and the linkage to household survey data and multiple occupational measures associated with SARS-CoV-2 exposure. However, there were several limitations. First, we used indirect measures of occupational risk based on primary lifetime occupation. Direct measures of occupational exposure would also eliminate the need to use detailed occupation codes and thereby support analyses of smaller racial and ethnic groups disproportionately affected by COVID-19. Second, we may underestimate the importance of occupation because we did not account for within-household transmission initiated by an occupational exposure.^30^ Third, we only included confirmed COVID-19 deaths, conceivably leading to an underestimate of true inequities: some racial and ethnic groups may be more likely to die at home without COVID-19 testing and, therefore, not be counted as a confirmed COVID-19 death.^38^ Other research has estimated all-cause excess deaths,^9,22^ but this was not possible here because of the small number of deaths occurring with strata jointly defined by race and ethnicity, education, occupation, and other covariates. Fourth, because of the inherent dependency between education and occupation, we could not fully disentangle education from other occupational factors. Fifth, we could only control for potential confounders measured in both the death and ACS records. Education and occupation may, therefore, be proxies for other factors such as intergenerational wealth and debt, parental education, and noncitizen legal status.^39^ Additional unmeasured factors stemming from structural racism likely mediate the association between race and ethnicity and COVID-19 mortality, such as housing composition and density, access to health care, and institutionalized residence. Combining death and ACS data introduces the possibility of differential misclassification between the 2 sources; in multivariate settings, the direction of potential bias is difficult to project.

## Conclusions

In this population-based cohort study of working-age California adults, educational attainment and occupation were important factors associated with risk for COVID-19 mortality. Future COVID-19 mitigation strategies should include policies, protections, and vigilant monitoring to protect workers in low-education, essential, on-site, and low-wage jobs from COVID-19 mortality. These steps are not a panacea, but they have the potential to save lives and reduce some racial and ethnic inequities in COVID-19 mortality.

## Notes

Supplement. **eTable 1.** Definition of California Regions **eAppendix 1.** Occupational Measures **eTable 2. **Occupation-Based Measures Associated With COVID-19 Exposure Risk **eAppendix 2.** Occupation Code Crosswalks **eAppendix 3.** Bootstrapped Confidence Intervals **eTable 3.** Estimated Change in COVID-19 Mortality per 100 000 People When Setting Educational Attainment and Occupational Characteristics to (a) the Education and Occupational Distribution of White People of the Same Gender or (b) the Lowest-Risk Educational or Occupational Position, by Race, Ethnicity, and Gender, for Individuals Aged 18-65, California, January 1, 2020 to February 12, 2021 **eTable 4.** Demographic, Educational, and Occupational Characteristics of Study Population by Race and Ethnicity **eTable 5.** Estimated COVID-19 Mortality Risks for Individuals Aged 18-65, per 100 000 Persons, by Race, Ethnicity, and Gender, if All Groups Had the Same Composition and Distribution of Occupational Characteristics as White People of the Same Gender, California, January 1, 2020 to February 12, 2021 **eTable 6.** Estimated COVID-19 Mortality Risks and Percent Change in Mortality Risk After Accounting for Racial and Ethnic Differences in Composition, Education, and Occupational Characteristics, for Individuals Aged 18-65, per 100 000 Persons, by Race, Ethnicity, and Gender, Sensitivity Analysis Results Based on Logistic Regression, California, January 1, 2020 to February 12, 2021
eReferences
