# Supplementary material for: Occupation and Educational Attainment Characteristics Associated With COVID-19 Mortality by Race and Ethnicity in California
Source: JAMA Netw Open. 2022 Apr 22;5(4):e228406. doi: 10.1001/jamanetworkopen.2022.8406 (PMC9034406; doi:10.1001/jamanetworkopen.2022.8406)
Supplement: Supplement. — eTable 1. Definition of California Regions eAppendix 1. Occupational Measures eTable 2. Occupation-Based Measures Associated With COVID-19 Exposure Risk eAppendix 2. Occupation Code Crosswalks eAppendix 3. Bootstrapped Confidence Intervals eTable 3. Estimated Change in COVID-19 Mortality per 100 000 People When Setting Educational Attainment and Occupational Characteristics to (a) the Education and Occupational Distribution of White People of the Same Gender or (b) the Lowest-Risk Educational or Occupational Position, by Race, Ethnicity, and Gender, for Individuals Aged 18-65, California, January 1, 2020 to February 12, 2021 eTable 4. Demographic, Educational, and Occupational Characteristics of Study Population by Race and Ethnicity eTable 5. Estimated COVID-19 Mortality Risks for Individuals Aged 18-65, per 100 000 Persons, by Race, Ethnicity, and Gender, if All Groups Had the Same Composition and Distribution of Occupational Characteristics as White People of the Same Gender, California, January 1, 2020 to February 12, 2021 eTable 6. Estimated COVID-19 Mortality Risks and Percent Change in Mortality Risk After Accounting for Racial and Ethnic Differences in Composition, Education, and Occupational Characteristics, for Individuals Aged 18-65, per 100 000 Persons, by Race, Ethnicity, and Gender, Sensitivity Analysis Results Based on Logistic Regression, California, January 1, 2020 to February 12, 2021 eReferences [file jamanetwopen-e228406-s001.pdf]

Supplemental Online Content

Matthay EC, Duchowny KA, Riley AR, et al. Occupation and educational attainment characteristics associated with COVID-19 mortality by race and ethnicity in California. *JAMA Netw Open*. 2022;5(4):e228406. doi:10.1001/jamanetworkopen.2022.8406

eTable 1. Definition of California Regions
eAppendix 1. Occupational Measures
eTable 2. Occupation-Based Measures Associated With COVID-19 Exposure Risk
eAppendix 2. Occupation Code Crosswalks
eAppendix 3. Bootstrapped Confidence Intervals
eTable 3. Estimated Change in COVID-19 Mortality per 100 000 People When Setting Educational Attainment and Occupational Characteristics to (a) the Education and Occupational Distribution of White People of the Same Gender or (b) the Lowest-Risk Educational or Occupational Position, by Race, Ethnicity, and Gender, for Individuals Aged 18-65, California, January 1, 2020 to February 12, 2021
eTable 4. Demographic, Educational, and Occupational Characteristics of Study Population by Race and Ethnicity
eTable 5. Estimated COVID-19 Mortality Risks for Individuals Aged 18-65, per 100 000 Persons, by Race, Ethnicity, and Gender, if All Groups Had the Same Composition and Distribution of Occupational Characteristics as White People of the Same Gender, California, January 1, 2020 to February 12, 2021
eTable 6. Estimated COVID-19 Mortality Risks and Percent Change in Mortality Risk After Accounting for Racial and Ethnic Differences in Composition, Education, and Occupational Characteristics, for Individuals Aged 18-65, per 100 000 Persons, by Race, Ethnicity, and Gender, Sensitivity Analysis Results Based on Logistic Regression, California, January 1, 2020 to February 12, 2021
eReferences

This supplemental material has been provided by the authors to give readers additional information about their work.

© 2022 Matthay EC et al. *JAMA Network Open.*

eTable 1: Definition of California Regions

Region number	Counties included
1	Butte, Colusa, Del Norte, El Dorado, Glenn, Lassen, Modoc, Nevada, Placer, Plumas, Sacramento, Shasta, Sierra, Siskiyou, Sutter, Tehama, Trinity, Yolo, Yuba
2	Humboldt, Lake, Mendocino, Napa, Sonoma
3	Alameda, Contra Costa, Marin, San Francisco, San Mateo, Santa Clara, Solano
4	Alpine, Amador, Calaveras, Madera, Mariposa, Merced, Mono, San Joaquin, Stanislaus, Tuolumne, Inyo
5	Monterey, San Benito, San Luis Obispo, Santa Barbara, Santa Cruz, Ventura
6	Fresno, Kern, Kings, Tulare
7	Riverside, San Bernardino
8	Los Angeles
9	Orange
10	Imperial, San Diego

© 2022 Matthay EC et al. *JAMA Network Open.*

eAppendix 1: Occupational Measures

We characterized occupations using multiple measures hypothesized to be related to SARS-CoV-2 exposure risk (Appendix Table 1). First, as in previous research,[1] a team of three researchers manually categorized the 529 unique 2010 Census occupation codes into 9 occupational sectors based on the California official definition of essential work[2] and retail work. The categories were: facilities, food/agriculture, government/community, health/emergency, manufacturing, retail, transportation/logistics, not essential, and unemployed/not in labor force/missing.

Second, we used the O*NET database to link occupation codes to characteristics of each occupation. Overseen by the Bureau of Labor statistics, O*NET is based on surveys completed by employees, employers, and job experts, and includes measures of required knowledge and skills, typical tasks, exposures encountered, and the workplace environment for nearly 1,000 occupations. We used 13 O*NET measures deemed relevant to COVID-19 exposure risk in previous research[3–6]—for example, the importance of assisting and caring for others, or the importance of working with computers (Appendix Table 2). We also considered Dingel and Neiman's classification of which jobs can be done at home during the COVID-19 pandemic (telework), which was based on a composite of O*NET measures.[5]

Third, individuals with lower incomes have less ability to not work or forgo income when faced with undesired COVID-19 exposure risk. We therefore characterized mean, median, 10th, 25th, 75th, and 90th percentiles of annual and hourly wages within each occupation code, based on the Bureau of Labor Statistics May 2019 report of wages for each occupation code. To merge the O*NET, telework, and wages measures to the death and population data, we used multiple available crosswalks from the occupational coding schemes used in each source to 2010 Census occupation codes (see Appendix "Occupation code crosswalks").

In our presentation of the results, we focus on the occupational measures of essential sector, telework, and median annual wages, because within the three major categories of measures (sector, O*NET-based measures, and wages) these measures were the strongest predictors of COVID-19 death in the study population. Results for the remaining occupational measures are presented in Appendix Table 5.

© 2022 Matthay EC et al. *JAMA Network Open.*

eTable 2: Occupation-Based Measures Related to COVID-19 Exposure Risk

Source	Measure	Description
California official essential worker designations[1]	9 categories of essential and non-essential worker	Facilities, food/agriculture, government/community, health/emergency, manufacturing, retail, transportation/logistics, not essential, and unemployed/missing
Dingel and Neiman, 2020[5]	Telework	Classification (0/1) of feasibility of working from home for each occupation. Composite measure based on 15 O*NET job measures.
Bureau of Labor Statistics May 2019 National Occupational Employment and Wage Estimates	Mean, median, 10th, 25th, 75th, and 90th percentiles of annual wages; Mean, median, 10th, 25th, 75th, and 90th percentiles of hourly wages	Estimates are produced by the Occupational Employment and Wage Statistics program and provide wage estimates annually for nearly 800 occupations. Estimates are calculated with data collected from employers in all industries in urban and rural areas nationwide.
O*NET 2019 surveys of employees, employers, and job experts	Importance of assisting and caring for others	Work activities: Importance of "providing personal assistance, medical attention, emotional support, or other personal care to others such as coworkers, customers, or patients". Examples of top ranking: Family medicine physicians, physical therapist assistants
	Level of assisting and caring for others	Work activities: Level of "providing personal assistance, medical attention, emotional support, or other personal care to others such as coworkers, customers, or patients". Examples of top ranking: Nurse anesthetists, sports medicine physicians
	Contact with others	Work context: "How much does this job require the worker to be in contact with others (face-to-face, by telephone, or otherwise) in order to perform it?" Examples of top ranking: Allergists and immunologists, education and childcare administrators at preschools and daycares, receptionists and information clerks, telemarketers
	Physical proximity	Work context: "To what extent does this job require the worker to perform job tasks in close physical proximity to other people?" Examples of top ranking: acupuncturists, choreographers, dental hygienists, sports medicine physicians
	Deal with physically aggressive people	Work context: "How frequently does this job require the worker to deal with physical aggression of violent individuals?" Examples of top ranking: First-line supervisors of correctional officers, correctional officers and jailers, police and sheriff's patrol officers, psychiatric aides
	Exposed to disease or infections	Work context: "How often does this job require exposure to disease/infections?" Examples of top ranking: Acute care nurses, dental hygienists, general internal medicine physicians, general pediatricians
	Face-to-face discussions	Work context: "How often do you have to have face-to-face discussions with individuals or teams in this job?" Amusement and recreational attendants, buyers and purchasing agents of farm products, chief executives, electrical power-line installers and repairers, forest and conservation technicians, neurologists
	Importance of interacting with computers	Work activities: Importance of "using computers and computer systems (including hardware and software) to program, write software, set up functions, enter data, or process information". Examples of top ranking: Computer systems engineers/architects, radio frequency identification device specialists, database administrators
	Level of interaction with computers	Work activities: Level of "using computers and computer systems (including hardware and software) to program, write software, set up functions, enter data, or process information". Examples of top ranking: Computer network architects, computer systems engineers/architects, database architects
	Importance of performing for or working directly with the public	Work activities: Importance of "performing for people or dealing directly with the public. This includes serving customers in restaurants and stores, and receiving clients or guests". Examples of top ranking: Flight attendants, morticians/undertakers and funeral arrangers, demonstrators and product promoters
	Level of performing for or working directly with the public	Work activities: Level of "performing for people or dealing directly with the public. This includes serving customers in restaurants and stores, and receiving clients or guests". Examples of top ranking: Prosthodontists, judges and magistrates, morticians/undertakers and funeral arrangers
	Wear specialized protective or safety equipment such as breathing apparatus, safety harness, full protection suits, or radiation protection	Work context: "How much does this job require wearing specialized protective or safety equipment such as breathing apparatus, safety harness, full protection suits, or radiation protection?" Examples of top ranking: Fiberglass laminators and fabricators, wind turbine service technicians, hazardous materials removal workers
	Work with group or team	Work context: "How important is it to work with others in a group or team in this job?" Examples of top ranking: Actors, family medicine physicians, airline pilots/copilots and flight engineers

© 2022 Matthay EC et al. *JAMA Network Open.*

eAppendix 2: Occupation Code Crosswalks

Death and American Community Survey records

Death records included open text fields for primary occupation and primary industry, described as "type of work done during most of working life". We used the National Institute for Occupational Safety and Health's Industry and Occupation Computerized Coding System, an automated machine-learning based system, to convert the open text fields for occupation and industry to standardized 2010 Census Codes (529 unique codes). To align the 2010 Census occupation codes in the death records with American Community Survey (ACS) which used 2018 Census occupation codes (570 unique codes), we used the Census crosswalk.[7] When two 2018 codes were assigned to the same 2010 code, we assigned them the same 2010 code. When one 2018 code was assigned to multiple 2010, we arbitrarily selected the first, recognizing that the ultimate occupational measures used in the analysis (telework, wages, etc.) would not distinguish between these sub-categories.

*O*NET data*

To merge the O*NET data to the death/ACS data, we applied multiple crosswalks: from 2019 O*NET SOC codes (1016 unique codes) to 2018 SOC codes (867 unique codes),[8] then from 2018 SOC codes to 2010 SOC codes (840 unique codes),[9] then from 2010 SOC codes to 2010 Census codes (529 unique codes).[7] At each stage, we applied the same procedures as above to assign codes that split or combined when transitioning from one coding scheme to the next.

Telework data

To merge the Dingel and Neiman telework data to the death/ACS data, we applied multiple crosswalks: from 2010 O*NET SOC codes (1110 unique codes) to 2010 SOC codes (840 unique codes),[10] then from 2010 SOC codes to 2010 Census codes (529 unique codes).[7] At each stage, we applied the same procedures to assign codes that split or combined when transitioning from one coding scheme to the next.

Wages data

To merge the US Bureau of Labor Statistics telework data to the death/ACS data, we applied multiple crosswalks: from the OES hybrid SOC coding scheme (824 unique codes) to 2010 SOC codes (840 unique codes),[11] then from 2010 SOC codes to 2010 Census codes (529 unique codes).[7] At each stage, we applied the same procedures to assign codes that split or combined when transitioning from one coding scheme to the next.

© 2022 Matthay EC et al. *JAMA Network Open.*

eAppendix 3: Bootstrapped Confidence Intervals

Confidence intervals were generating using the nonparametric bootstrap.[12] The analytic data were at the stratum-level with weights corresponding the number of people represented in each row, but the relevant unit of analysis was the person. Thus, to create each bootstrapped dataset, we expanded the analytic data by duplicating each row by the number of persons represented by that row, then sampled rows with replacement from the expanded dataset, and collapsed back to the stratum level for analysis.

Because the data represented 25 million people and were stored on a secure server with limited computational capacity, it was not feasible to bootstrap the data extensively (e.g. 10,000 times). We tested small numbers of bootstraps incrementally (5, 10, 20, 40, 80, 100, 125, 150) and defined convergence of the standard errors of the parameter estimates as the point at which the absolute value of the change in the SE estimate was less than 0.5, where 0.5 was selected as an amount below which there would be no difference in the substantive implications of the results. In practice, this level of convergence was achieved for all parameters after 125 bootstraps.

eTable 3: Estimated Change in COVID-19 Mortality per 100,000 People When Setting Educational Attainment and Occupational Characteristics to (a) the Education and Occupational Distribution of White People of the Same Gender or (b) the Lowest-Risk Educational or Occupational Position, by Race, Ethnicity, and Gender, for Individuals Aged 18-65, California, January 1, 2020-February 12, 2021

Mediator	Race/ethnicity	Change in COVID-19 mortality, if all individuals had the educational attainment and occupational characteristics of White people of the same gender (95% CI)		Change in COVID-19 mortality, if all individuals held the lowest-risk educational and occupational position (95% CI)	
		**Men**	**Women**	**Men**	**Women**
Education	Asian	6 (5, 7)	3 (2, 4)	-10 (-14, -6)	-4 (-6, -2)
	Black	-10 (-12, -7)	-4 (-6, -2)	-41 (-50, -31)	-19 (-26, -12)
	Latinx	-24 (-27, -22)	-16 (-17, -14)	-56 (-62, -49)	-35 (-38, -31)
	White	(ref)	(ref)	-11 (-13, -10)	-9 (-10, -8)
	Other	-6 (-8, -4)	-4 (-5, -2)	-34 (-44, -23)	-23 (-30, -17)
Essential sector	Asian	-1 (-2, 1)	-1 (-2, 0)	-9 (-14, -4)	-1 (-4, 2)
	Black	9 (6, 12)	1 (0, 2)	8 (-3, 19)	-3 (-10, 5)
	Latinx	-13 (-14, -11)	-2 (-2, -1)	-38 (-44, -32)	-11 (-15, -8)
	White	(ref)	(ref)	-10 (-12, -8)	-2 (-4, -1)
	Other	-1 (-3, 0)	0 (-1, 0)	-23 (-33, -14)	-7 (-14, 1)
Not Telework	Asian	-1 (-2, -1)	0 (0, 0)	-7 (-12, -3)	0 (-2, 3)
	Black	10 (7, 12)	1 (0, 2)	15 (5, 24)	3 (-3, 9)
	Latinx	-9 (-10, -8)	-2 (-3, -2)	-23 (-28, -18)	-11 (-14, -8)
	White	(ref)	(ref)	-8 (-10, -6)	-2 (-4, -1)
	Other	-1 (-3, 0)	-1 (-1, 0)	-11 (-20, -2)	-9 (-14, -3)
Annual wage	Asian	0 (-1, 0)	0 (0, 1)	-13 (-18, -7)	-3 (-7, 1)
	Black	2 (-2, 5)	2 (0, 3)	-5 (-20, 11)	16 (-1, 32)
	Latinx	-13 (-15, -11)	-4 (-5, -3)	-38 (-46, -29)	-24 (-31, -17)
	White	(ref)	(ref)	-9 (-12, -7)	-4 (-6, -2)
	Other	-2 (-4, 0)	-1 (-2, 0)	-28 (-39, -16)	-6 (-18, 6)
All occupational factors	Asian	0 (-2, 2)	-1 (-2, 0)	-15 (-21, -9)	-7 (-12, -3)
	Black	1 (-4, 6)	-1 (-3, 2)	-10 (-28, 9)	10 (-8, 28)
	Latinx	-18 (-20, -15)	-5 (-6, -3)	-55 (-64, -46)	-28 (-36, -20)
	White	(ref)	(ref)	-13 (-16, -10)	-6 (-9, -4)
	Other	-2 (-4, 0)	-2 (-3, -1)	-35 (-49, -21)	-10 (-22, 3)
Education and all occupational factors	Asian	6 (4, 8)	3 (2, 4)	-18 (-25, -12)	-10 (-14, -5)
	Black	0 (-4, 4)	-3 (-6, -1)	-22 (-39, -6)	-4 (-22, 14)
	Latinx	-31 (-34, -28)	-16 (-18, -15)	-75 (-85, -66)	-48 (-54, -42)
	White	(ref)	(ref)	-17 (-20, -13)	-12 (-15, -9)
	Other	-3 (-6, -1)	-4 (-5, -3)	-42 (-56, -28)	-23 (-35, -10)

Legend: “Other” race and ethnicity includes American Indian, Alaskan Native, Native Hawaiians, other Pacific Islanders, multi-race, and unspecified (all non-Latinx). All models are adjusted for age group, USA nativity, and California region of residence. Risk differences were calculated by comparing the composition-adjusted COVID-19 mortality risk (the COVID-19 mortality risk if all racial/ethnic groups had the same distribution of covariates as Whites) to the

© 2022 Matthay EC et al. *JAMA Network Open.*

composition- and mediator-adjusted COVID-19 mortality risk (the COVID-19 mortality risk if all racial/ethnic groups had the same distribution of covariates and education/occupation mediator(s) as Whites, or the COVID-19 mortality risk if all individuals held the safest educational and/or occupational position [Bachelor's degree or higher, non-essential, telework-able, highest quintile of median annual wages]).

© 2022 Matthay EC et al. *JAMA Network Open.*

eTable 4: Demographic, Educational, and Occupational Characteristics of Study Population by Race and Ethnicity

	**Asian**		**Black**		**Latinx**		**White**		**Other**	
	N	***%***	***N***	***%***	***N***	***%***	***N***	***%***	***N***	***%***
Persons	3,925,494	16%	1,472,151	6%	9,859,259	39%	9,040,379	36%	937,809	4%
COVID-19 death										
No	3,924,376	100%	1,471,185	100%	9,849,030	100%	9,038,381	100%	937,337	100%
Yes	1,118	0%	966	0%	10,229	0%	1,998	0%	472	0%
Age (years)										
18-24	472,163	12%	226,881	15%	1,799,211	18%	1,017,420	11%	172,745	18%
25-44	1,818,665	46%	639,966	43%	4,717,922	48%	3,702,330	41%	468,423	50%
45-65	1,634,666	42%	605,304	41%	3,342,126	34%	4,320,629	48%	296,641	32%
Gender										
Women	2,063,142	53%	723,166	49%	4,852,961	49%	4,407,412	49%	468,250	50%
Men	1,862,352	47%	748,985	51%	5,006,298	51%	4,632,967	51%	469,559	50%
Foreign-born										
No	1,181,970	30%	1,319,700	90%	5,576,110	57%	8,078,201	89%	773,811	83%
Yes	2,743,509	70%	152,429	10%	4,283,083	43%	962,108	11%	163,934	17%
Missing	15	0%	22	0%	66	0%	70	0%	64	0%
Educational attainment										
No high school degree and no GED	295,891	8%	136,774	9%	2,726,451	28%	402,555	4%	71,527	8%
High school degree or GED	536,599	14%	369,080	25%	2,815,628	29%	1,676,499	19%	196,998	21%
Some college or Associate’s degree	946,735	24%	592,886	40%	2,993,868	30%	3,006,774	33%	342,069	36%
Bachelor’s degree or beyond	2,146,241	55%	373,367	25%	1,322,962	13%	3,954,439	44%	327,154	35%
Missing	28	0%	44	0%	350	0%	112	0%	61	0%
Worker sector										
Facilities	169,041	4%	81,139	6%	1,508,582	15%	733,589	8%	69,032	7%
Food and agriculture	235,104	6%	63,386	4%	1,065,046	11%	466,679	5%	58,329	6%
Government and community	303,395	8%	166,907	11%	748,296	8%	1,000,243	11%	94,959	10%
Health or emergency	440,885	11%	163,842	11%	602,333	6%	733,815	8%	84,654	9%
Manufacturing	175,261	4%	33,570	2%	616,232	6%	302,820	3%	29,690	3%
Retail	199,368	5%	99,151	7%	720,072	7%	501,385	6%	63,657	7%
Transportation and logistics	177,834	5%	145,460	10%	957,648	10%	455,806	5%	68,379	7%
Not essential	1,623,780	41%	386,578	26%	1,889,471	19%	3,582,906	40%	322,266	34%
Unemployed or missing	600,826	15%	332,118	23%	1,751,579	18%	1,263,136	14%	146,843	16%
Telework-able occupation										
Yes	1,683,540	43%	484,019	33%	2,262,639	23%	4,159,608	46%	368,002	39%
No	1,582,613	40%	625,829	43%	5,716,117	58%	3,444,972	38%	404,135	43%
Unemployed/not in labor force	600,826	15%	332,118	23%	1,751,573	18%	1,263,135	14%	146,843	16%
Missing	58,515	1%	30,185	2%	128,930	1%	172,664	2%	18,829	2%
Median annual wage for occupation										
$22,200 – 29,000	583,668	15%	275,161	19%	2,544,448	26%	1,151,490	13%	167,442	18%
$29,001 – 39,100	477,281	12%	294,545	20%	2,065,638	21%	1,136,431	13%	156,869	17%
$39,101 – 51,700	521,040	13%	201,257	14%	1,644,828	17%	1,442,786	16%	139,735	15%

© 2022 Matthay EC et al. *JAMA Network Open.*

$51,701 – 73,800	666,910	17%	203,665	14%	1,104,127	11%	1,839,359	20%	155,091	17%
$73,800 +	1,048,661	27%	147,163	10%	712,908	7%	2,124,201	23%	164,032	17%
Unemployed/not in labor force	600,826	15%	332,118	23%	1,751,573	18%	1,263,135	14%	146,843	16%
Missing	27,108	1%	18,242	1%	35,737	0%	82,977	1%	7,797	1%

Legend: "Other" race and ethnicity includes American Indian, Alaskan Native, Native Hawaiians, other Pacific Islanders, multi-race, and unspecified (all non-Latinx).

© 2022 Matthay EC et al. *JAMA Network Open.*

eTable 5: Estimated COVID-19 Mortality Risks for Individuals Aged 18-65, per 100,000 Persons, by Race, Ethnicity, and Gender, if All Groups Had the Same Composition and Distribution of Occupational Characteristics as White People of the Same Gender, California, January 1, 2020-February 12, 2021

		Men		Women	
**Occupational measure (mediator)**	**Race/ ethnicity**	**Composition-adjusted (95% CI)**	**Composition- and mediator- adjusted (95% CI)**	**Composition-adjusted (95% CI)**	**Composition- and mediator- adjusted (95% CI)**
Importance of assisting and caring for others	Asian	27 (21, 33)	26 (20, 33)	13 (9, 16)	14 (10, 17)
	Black	87 (79, 95)	101 (92, 110)	51 (44, 58)	52 (45, 59)
	Latinx	142 (137, 147)	145 (140, 150)	70 (66, 74)	70 (66, 74)
	White	(ref)	(ref)	(ref)	(ref)
	Other	84 (74, 94)	87 (77, 98)	46 (36, 56)	46 (37, 56)
Level of assisting and caring for others	Asian	27 (21, 34)	26 (20, 33)	13 (10, 16)	14 (10, 17)
	Black	87 (79, 95)	100 (91, 109)	51 (44, 58)	52 (45, 59)
	Latinx	142 (136, 148)	142 (137, 148)	70 (66, 73)	69 (66, 73)
	White	(ref)	(ref)	(ref)	(ref)
	Other	84 (73, 94)	87 (76, 97)	46 (38, 54)	46 (38, 54)
Contact with others	Asian	27 (21, 33)	27 (20, 33)	13 (9, 16)	14 (10, 17)
	Black	87 (78, 95)	99 (89, 109)	51 (44, 58)	54 (47, 61)
	Latinx	142 (137, 147)	143 (138, 148)	70 (66, 74)	68 (64, 72)
	White	(ref)	(ref)	(ref)	(ref)
	Other	84 (74, 94)	87 (77, 98)	46 (37, 55)	46 (37, 55)
Physical proximity	Asian	27 (21, 33)	26 (20, 32)	13 (9, 17)	12 (8, 16)
	Black	87 (78, 96)	97 (87, 107)	51 (44, 58)	53 (46, 60)
	Latinx	142 (138, 147)	143 (138, 147)	70 (66, 73)	69 (65, 73)
	White	(ref)	(ref)	(ref)	(ref)
	Other	83 (73, 94)	85 (74, 96)	46 (37, 55)	46 (36, 55)
Deal with physically aggressive people	Asian	28 (22, 34)	26 (20, 33)	13 (9, 16)	13 (10, 17)
	Black	87 (77, 96)	98 (87, 108)	50 (44, 57)	50 (43, 57)
	Latinx	142 (137, 148)	144 (138, 150)	70 (66, 74)	69 (65, 73)
	White	(ref)	(ref)	(ref)	(ref)
	Other	84 (73, 94)	85 (75, 96)	46 (38, 54)	45 (37, 54)
Exposed to disease or infections	Asian	30 (23, 37)	28 (21, 35)	13 (10, 17)	14 (10, 17)
	Black	88 (78, 97)	101 (90, 112)	51 (44, 58)	52 (45, 58)
	Latinx	142 (137, 147)	144 (139, 150)	70 (66, 74)	69 (65, 73)
	White	(ref)	(ref)	(ref)	(ref)
	Other	86 (75, 97)	91 (79, 102)	47 (39, 55)	47 (39, 56)
Face-to-face discussions	Asian	27 (21, 34)	27 (20, 33)	13 (9, 16)	12 (9, 16)
	Black	87 (78, 95)	97 (87, 106)	51 (45, 57)	52 (45, 58)
	Latinx	142 (137, 147)	143 (138, 148)	70 (66, 73)	68 (65, 72)
	White	(ref)	(ref)	(ref)	(ref)
	Other	84 (73, 95)	87 (76, 98)	46 (38, 54)	46 (37, 54)
Importance of interacting with computers	Asian	28 (21, 34)	28 (21, 34)	13 (10, 16)	13 (10, 16)
	Black	86 (77, 95)	93 (83, 102)	50 (43, 58)	51 (43, 58)
	Latinx	142 (137, 147)	132 (127, 138)	70 (66, 73)	67 (63, 71)
	White	(ref)	(ref)	(ref)	(ref)
	Other	83 (72, 95)	82 (71, 93)	46 (38, 54)	46 (38, 54)
Level of interaction with computers	Asian	26 (20, 32)	26 (21, 32)	12 (8, 16)	12 (8, 16)
	Black	85 (77, 93)	93 (83, 102)	48 (41, 55)	49 (42, 56)
	Latinx	142 (137, 148)	136 (130, 142)	67 (63, 71)	65 (61, 69)
	White	(ref)	(ref)	(ref)	(ref)
	Other	85 (74, 97)	85 (73, 96)	44 (35, 54)	44 (34, 53)
Importance of performing for or working directly with the public	Asian	27 (21, 34)	27 (21, 34)	13 (9, 17)	13 (9, 17)
	Black	87 (78, 96)	99 (89, 109)	51 (45, 57)	53 (46, 59)
	Latinx	142 (137, 148)	142 (137, 148)	70 (66, 74)	69 (65, 73)
	White	(ref)	(ref)	(ref)	(ref)
	Other	84 (73, 95)	88 (76, 99)	46 (37, 55)	46 (38, 55)
Level of performing for or working directly with the public	Asian	27 (21, 34)	28 (21, 35)	13 (9, 16)	13 (10, 17)
	Black	86 (78, 95)	99 (89, 108)	51 (44, 59)	53 (45, 61)
	Latinx	140 (134, 145)	140 (135, 145)	70 (67, 74)	70 (66, 73)
	White	(ref)	(ref)	(ref)	(ref)
	Other	82 (71, 92)	85 (74, 96)	46 (36, 55)	46 (36, 55)

© 2022 Matthay EC et al. *JAMA Network Open.*

Wear specialized protective or safety equipment such as breathing apparatus, safety harness, full protection suits, or radiation protection	Asian	28 (22, 35)	29 (22, 35)	14 (10, 18)	13 (9, 17)
	Black	92 (82, 102)	109 (97, 121)	51 (43, 59)	52 (44, 60)
	Latinx	144 (138, 150)	144 (138, 150)	72 (67, 76)	71 (66, 75)
	White	(ref)	(ref)	(ref)	(ref)
	Other	86 (74, 98)	88 (76, 101)	47 (37, 56)	46 (36, 56)
Work with group or team	Asian	27 (21, 33)	27 (21, 33)	13 (9, 16)	13 (9, 16)
	Black	87 (78, 95)	96 (87, 105)	51 (44, 58)	54 (47, 61)
	Latinx	142 (137, 147)	139 (134, 144)	70 (66, 74)	69 (65, 73)
	White	(ref)	(ref)	(ref)	(ref)
	Other	84 (73, 95)	86 (74, 98)	46 (37, 55)	46 (36, 56)
Hourly wages: mean	Asian	27 (22, 32)	27 (22, 33)	13 (9, 18)	14 (10, 18)
	Black	87 (79, 96)	90 (80, 99)	51 (44, 59)	53 (45, 60)
	Latinx	143 (137, 149)	131 (125, 137)	71 (67, 75)	67 (64, 71)
	White	(ref)	(ref)	(ref)	(ref)
	Other	83 (73, 94)	81 (71, 92)	46 (37, 54)	45 (36, 53)
Hourly wages: 10th percentile	Asian	27 (22, 33)	28 (22, 34)	13 (10, 17)	14 (10, 18)
	Black	87 (79, 95)	89 (81, 98)	51 (44, 58)	54 (46, 61)
	Latinx	143 (138, 148)	131 (126, 136)	71 (67, 75)	68 (64, 72)
	White	(ref)	(ref)	(ref)	(ref)
	Other	83 (73, 93)	82 (72, 91)	46 (37, 55)	46 (37, 54)
Hourly wages: 25th percentile	Asian	27 (21, 33)	27 (21, 33)	14 (9, 18)	14 (10, 18)
	Black	88 (79, 97)	89 (80, 99)	52 (44, 59)	53 (45, 61)
	Latinx	144 (138, 150)	132 (126, 138)	71 (68, 75)	68 (64, 72)
	White	(ref)	(ref)	(ref)	(ref)
	Other	84 (74, 94)	82 (72, 92)	47 (37, 56)	46 (36, 55)
Hourly wages: Median	Asian	27 (22, 33)	27 (22, 33)	13 (10, 17)	14 (10, 18)
	Black	87 (79, 95)	90 (81, 99)	51 (44, 58)	53 (45, 61)
	Latinx	143 (138, 148)	132 (126, 137)	71 (67, 75)	67 (63, 71)
	White	(ref)	(ref)	(ref)	(ref)
	Other	83 (73, 93)	82 (72, 91)	46 (37, 55)	45 (37, 54)
Hourly wages: 75th percentile	Asian	27 (22, 32)	27 (22, 33)	13 (9, 18)	14 (10, 18)
	Black	87 (79, 96)	89 (80, 98)	51 (44, 59)	53 (45, 60)
	Latinx	143 (137, 149)	131 (125, 137)	71 (67, 75)	67 (64, 71)
	White	(ref)	(ref)	(ref)	(ref)
	Other	83 (73, 94)	81 (70, 91)	46 (37, 54)	45 (36, 53)
Hourly wages: 90th percentile	Asian	27 (22, 32)	27 (21, 32)	13 (9, 18)	14 (10, 18)
	Black	87 (79, 96)	91 (82, 100)	51 (44, 59)	53 (45, 60)
	Latinx	143 (137, 149)	131 (125, 137)	71 (67, 75)	67 (64, 71)
	White	(ref)	(ref)	(ref)	(ref)
	Other	83 (73, 94)	81 (71, 92)	46 (37, 54)	45 (36, 53)
Annual wages: mean	Asian	27 (21, 34)	27 (21, 34)	14 (10, 18)	14 (10, 18)
	Black	87 (78, 96)	88 (79, 98)	51 (45, 58)	52 (45, 59)
	Latinx	142 (136, 147)	128 (123, 134)	71 (67, 75)	67 (62, 71)
	White	(ref)	(ref)	(ref)	(ref)
	Other	83 (72, 94)	80 (70, 91)	44 (35, 53)	43 (34, 52)
Annual wages: 10th percentile	Asian	28 (22, 33)	28 (23, 34)	14 (11, 17)	14 (11, 18)
	Black	87 (79, 95)	89 (80, 98)	51 (44, 58)	53 (45, 60)
	Latinx	141 (136, 147)	130 (124, 135)	70 (66, 74)	67 (63, 71)
	White	(ref)	(ref)	(ref)	(ref)
	Other	82 (72, 93)	81 (70, 92)	45 (36, 53)	44 (35, 53)
Annual wages: 25th percentile	Asian	28 (21, 34)	28 (21, 34)	14 (10, 18)	14 (11, 18)
	Black	87 (78, 96)	89 (78, 100)	52 (44, 59)	52 (45, 60)
	Latinx	142 (137, 148)	130 (125, 135)	71 (67, 75)	67 (63, 71)
	White	(ref)	(ref)	(ref)	(ref)
	Other	84 (73, 95)	82 (71, 92)	45 (37, 53)	44 (36, 52)
Annual wages: 75th percentile	Asian	27 (21, 34)	27 (21, 34)	14 (10, 18)	14 (10, 18)
	Black	87 (78, 96)	88 (78, 97)	51 (45, 58)	52 (45, 59)
	Latinx	142 (136, 147)	129 (123, 134)	71 (67, 75)	67 (62, 71)
	White	(ref)	(ref)	(ref)	(ref)
	Other	83 (72, 94)	80 (69, 91)	44 (35, 53)	43 (34, 52)
Annual wages: 90th percentile	Asian	27 (21, 34)	27 (21, 34)	14 (10, 18)	14 (10, 18)
	Black	87 (78, 96)	90 (80, 99)	51 (45, 58)	52 (45, 59)
	Latinx	142 (136, 147)	129 (123, 134)	71 (67, 75)	67 (63, 71)
	White	(ref)	(ref)	(ref)	(ref)
	Other	83 (72, 94)	80 (70, 91)	44 (35, 53)	43 (34, 52)

© 2022 Matthay EC et al. *JAMA Network Open.*

eTable 6: Estimated COVID-19 Mortality Risks and Percent Change in Mortality Risk After Accounting for Racial and Ethnic Differences in Composition, Education, and Occupational Characteristics, for Individuals Aged 18-65, per 100,000 Persons, by Race, Ethnicity, and Gender, Sensitivity Analysis Results Based on Logistic Regression, California, January 1, 2020-February 12, 2021

Gender, race/ ethnicity	Unadjusted	Adjusted for composition (95% CI)	Adjusted for composition and work sector (95% CI)	Adjusted for composition and telework (95% CI)	Adjusted for composition- and wages (95% CI)	Adjusted for composition and education (95% CI)	Adjusted for composition and all occupational characteristics (work sector, telework, and wages) (95% CI)	Adjusted for composition, education and all occupational characteristics (work sector, telework, and wages) (95% CI)
Women								
Asian	15	18 (12, 23)	17 (12, 22)	16 (12, 21)	18 (13, 23)	20 (14, 26)	17 (-3260, 3294)	19 (-47, 86)
Black	48	51 (42, 60)	52 (38, 66)	53 (41, 64)	61 (9, 112)	49 (29, 70)	59 (-3090, 3208)	81 (-14, 176)
Latinx	60	63 (60, 67)	63 (60, 67)	61 (57, 66)	61 (56, 65)	47 (44, 50)	62 (-1971, 2094)	49 (41, 57)
White	15	(ref)	(ref)	(ref)	(ref)	(ref)	(ref)	(ref)
Other	29	45 (37, 54)	46 (36, 55)	47 (36, 57)	45 (35, 54)	41 (33, 49)	49 (-2378, 2475)	47 (36, 57)
Men								
Asian	41	48 (40, 57)	49 (40, 58)	48 (40, 57)	49 (40, 58)	55 (45, 65)	49 (39, 59)	54 (43, 65)
Black	77	87 (79, 96)	103 (92, 113)	101 (91, 112)	91 (81, 102)	77 (69, 85)	97 (84, 109)	99 (81, 117)
Latinx	138	122 (117, 128)	111 (106, 117)	115 (109, 120)	111 (105, 116)	100 (95, 106)	111 (105, 118)	99 (92, 105)
White	26	(ref)	(ref)	(ref)	(ref)	(ref)	(ref)	(ref)
Other	57	85 (75, 96)	86 (75, 97)	84 (74, 94)	83 (72, 94)	80 (70, 89)	89 (75, 102)	88 (75, 100)

Legend: “Other” race and ethnicity includes American Indian, Alaskan Native, Native Hawaiians, other Pacific Islanders, multi-race, and unspecified (all non-Latinx). Composition-adjusted COVID-19 mortality risks indicate the estimated COVID-19 mortality risk if all racial/ethnic groups had the same distribution of age, nativity, and region of residence as White people. Composition- and education-, work sector-, telework-, or wages-adjusted COVID-19 mortality risks indicate the estimated COVID-19 mortality risk if all racial/ethnic groups had the same distribution of age, nativity, region of residence, and educational attainment, work sector, telework capacity, or wages as White people, respectively.

© 2022 Matthay EC et al. *JAMA Network Open.*

eReferences

1 Chen Y-H, Glymour M, Riley A, *et al*. Excess mortality associated with the COVID-19 pandemic among Californians 18–65 years of age, by occupational sector and occupation: March through November 2020. *PLOS ONE* 2021; **16**: e0252454.

2 State of California. Essential workforce. 2020. https://covid19.ca.gov/essential-workforce/ (accessed July 6, 2021).

3 Hawkins D. Differential occupational risk for COVID-19 and other infection exposure according to race and ethnicity. *American Journal of Industrial Medicine* 2020; **63**: 817–20.

4 Baker MG. Nonrelocatable Occupations at Increased Risk During Pandemics: United States, 2018. *Am J Public Health* 2020; **110**: 1126–32.

5 Dingel JI, Neiman B. How many jobs can be done at home? *Journal of Public Economics* 2020; **189**: 104235.

6 Baker MG. Who cannot work from home? Characterizing occupations facing increased risk during the COVID-19 pandemic using 2018 BLS data. *medRxiv* 2020; : 2020.03.21.20031336.

7 US Census Bureau. 2018 Census Occupation Code List with Crosswalk. 2020; published online Aug 31. https://www2.census.gov/programs-surveys/demo/guidance/industry-occupation/2018-occupation-code-list-and-crosswalk.xlsx (accessed July 21, 2021).

8 O*NET Resource Center. Crosswalk O*NET-SOC 2019 to 2018 SOC - Occupational Listings at O*NET Resource Center. https://www.onetcenter.org/taxonomy/2019/soc.html (accessed July 21, 2021).

9 US Bureau of Labor Statistics. Crosswalk from the 2010 SOC to the 2018 SOC. 2017; published online Dec 6. https://www.bls.gov/soc/2018/soc_2010_to_2018_crosswalk.xlsx (accessed July 21, 2021).

10 O*NET Resource Center. Updating the O*NET-SOC Taxonomy: Incorporating the 2010 SOC Structure at O*NET Resource Center. 2010; published online Dec. https://www.onetcenter.org/reports/Taxonomy2010.html (accessed July 21, 2021).

11 US Bureau of Labor Statistics. OES 2019 Hybrid Structure. 2020; published online March 31. https://www.bls.gov/oes/oes_2019_hybrid_structure.xlsx.

12 Efron B, Tibshirani R. Bootstrap Methods for Standard Errors, Confidence Intervals, and Other Measures of Statistical Accuracy. *Statistical Science* 1986; **1**: 54–75.

© 2022 Matthay EC et al. *JAMA Network Open.*
